# Supplementary material for: The effects of problem-based learning (PBL) on undergraduate medical students’ critical thinking and communication skills development: A scoping review across resource-rich and resource-limited settings (2015–2024)
Source: PLoS One. 2026 Feb 13;21(2):e0342599. doi: 10.1371/journal.pone.0342599 (PMC12904385; doi:10.1371/journal.pone.0342599)
Supplement: S1 File — (DOCX) [file pone.0342599.s001.docx]

**Preferred Reporting Items for Systematic reviews and Meta-Analyses extension for Scoping Reviews (PRISMA-ScR) Checklist**

| **SECTION** | **ITEM** | **PRISMA-ScR CHECKLIST ITEM** | **REPORTED ON PAGE #** |
| --- | --- | --- | --- |
| **TITLE** | | | |
| Title | 1 | Reported on page 1: Title page and abstract title line  The title explicitly identifies the study as a scoping review | **Reported on page 1**  The effects of problem-based learning (PBL) on undergraduate medical students' critical thinking and communication skills development: A scoping review across resource-rich and resource-limited settings (2015-2024) |
| **ABSTRACT** | | | |
| Structured summary | 2 | Provide a structured summary that includes (as applicable): background, objectives, eligibility criteria, sources of evidence, charting methods, results, and conclusions that relate to the review questions and objectives. | **Reported on page 1&2**  The abstract includes background, objectives, eligibility criteria, information sources (Google Scholar, PubMed, ProQuest, ERIC), charting method, number of included studies (n = 8), and conclusions on perceived effects of PBL on critical thinking and communication skills. |
| **INTRODUCTION** | | | |
| Rationale | 3 | Describe the rationale for the review in the context of what is already known. Explain why the review questions/objectives lend themselves to a scoping review approach. | **Reported on page 3**  The manuscript situates the review within existing literature on PBL and identifies gaps related to early undergraduate medical training, geographic underrepresentation, and limited synthesis across resource-rich and resource-limited settings. A scoping approach is justified to map the breadth and characteristics of the evidence base. |
| Objectives | 4 | Provide an explicit statement of the questions and objectives being addressed with reference to their key elements (e.g., population or participants, concepts, and context) or other relevant key elements used to conceptualize the review questions and/or objectives. | **Reported on page 6**  This scoping review maps available evidence on the effects of PBL on critical thinking and  communication skills in undergraduate medical education, focusing on first-year medical students. It also seeks to compare PBL’s effectiveness in resource-rich and resource-limited settings |
| **METHODS** | | | |
| Protocol and registration | 5 | Indicate whether a review protocol exists; state if and where it can be accessed (e.g., a Web address); and if available, provide registration information, including the registration number. | **Reported on page 7**  The review followed Arksey and O’Malley’s framework but was not formally registered |
| Eligibility criteria | 6 | Specify characteristics of the sources of evidence used as eligibility criteria (e.g., years considered, language, and publication status), and provide a rationale. | **Reported on page 7**  Inclusion: (1) Focus on PBL; (2) Undergraduate medical students; (3) Outcomes: critical thinking or communication skills; (4) Published 2015-2024 in peer-reviewed journals. Exclusion: non-English studies, conference papers, grey literature, systematic reviews |
| Information sources* | 7 | Describe all information sources in the search (e.g., databases with dates of coverage and contact with authors to identify additional sources), as well as the date the most recent search was executed. | **Reported on Page 8**  Google Scholar, PubMed, ProQuest, and ERIC were searched. A snowballing approach was used to review the reference lists of included studies. |
| Search strategy | 8 | Present the full electronic search strategy for at least 1 database, including any limits used, such that it could be repeated. | **Reported on page 8**  Search terms: ‘Problem-Based Learning’ AND ‘Critical Thinking Skills,’ ‘Problem-Based Learning’ AND ‘Communication Skills,’ ‘Problem-Based Learning’ AND ‘Medical Education.’ Boolean operators (AND, OR) were used |
| Selection of sources of evidence† | 9 | State the process for selecting sources of evidence (i.e., screening and eligibility) included in the scoping review. | **Reported on page 9**  Studies underwent title and abstract screening, followed by full-text review by two independent reviewers. Disagreements were resolved through discussion. |
| Data charting process‡ | 10 | Describe the methods of charting data from the included sources of evidence (e.g., calibrated forms or forms that have been tested by the team before their use, and whether data charting was done independently or in duplicate) and any processes for obtaining and confirming data from investigators. | **Reported on page 15**  A standardized data extraction table was used, including study year, location, objectives, sample size, methodology, intervention, and outcomes. |
| Data items | 11 | List and define all variables for which data were sought and any assumptions and simplifications made. | **Reported on page 15**  Data extracted included author, year, country, study design, participant group, sample size, PBL intervention characteristics, and reported outcomes related to critical thinking and communication skills. Outcomes were treated as self-reported or measured as presented in each study, without standardization across instruments. |
| Critical appraisal of individual sources of evidence§ | 12 | If done, provide a rationale for conducting a critical appraisal of included sources of evidence; describe the methods used and how this information was used in any data synthesis (if appropriate). | **Reported on page 10**  No critical appraisal was conducted, as the aim was to map existing evidence rather than assess study quality. |
| Synthesis of results | 13 | Describe the methods of handling and summarizing the data that were charted. | **Reported on page 20**  A thematic synthesis approach was used. Findings were summarized descriptively, highlighting trends and variations across different educational settings. |
| **RESULTS** | | | |
| Selection of sources of evidence | 14 | Give numbers of sources of evidence screened, assessed for eligibility, and included in the review, with reasons for exclusions at each stage, ideally using a flow diagram. | **Reported on page 11-12**  21,001 studies identified; after screening, 8 met inclusion criteria. A PRISMA flow diagram illustrates the selection process. |
| Characteristics of sources of evidence | 15 | For each source of evidence, present characteristics for which data were charted and provide the citations. | **Reported on page 20**  Studies covered multiple countries, mainly in Asia. Most used cross-sectional study designs, with sample sizes ranging from 52 to 464 participants. |
| Critical appraisal within sources of evidence | 16 | If done, present data on critical appraisal of included sources of evidence (see item 12). | **Reported on page 10**  Not applicable, as no formal critical appraisal was conducted.” |
| Results of individual sources of evidence | 15 | For each included source of evidence, present the relevant data that were charted that relate to the review questions and objectives. | **Reported on page 15**  Table 1 presents author, year, country, design, sample size, intervention characteristics, and outcomes for all eight studies. Most studies used cross-sectional, questionnaire-based designs. Reported outcomes indicate perceived improvements in critical thinking, problem-solving, teamwork, and communication. Only one study focused exclusively on first-year medical students and assessed perceptions rather than objective performance measures |
| Synthesis of results | 18 | Summarize and/or present the charting results as they relate to the review questions and objectives. | The synthesis indicates that PBL is associated with perceived improvements in critical thinking and communication skills across both resource-rich and resource-limited settings. Evidence is methodologically heterogeneous, largely perception-based, and geographically limited to Asia, with only one study addressing first-year medical students and no studies from Africa.students and now is from Africa and other parts of the world. |
| **DISCUSSION** | | | |
| Summary of evidence | 19 | Summarize the main results (including an overview of concepts, themes, and types of evidence available), link to the review questions and objectives, and consider the relevance to key groups. | **Reported on page 32**  Eight studies on PBL in undergraduate medical students show consistent gains in critical thinking, problem-solving, teamwork, and communication. PBL was effective in both resource-rich and resource-limited settings, though first-year students may face adaptation challenges. Methodological heterogeneity and limited consideration of cultural or educational factors restrict generalizability. Findings guide educators, curriculum designers, and policy makers, while highlighting the need for standardized, multi-center, and contextually adapted PBL research. |
| Limitations | 20 | Discuss the limitations of the scoping review process. | **Reported on page 32**  Limitations include variability in study design, lack of standard assessment tools, and absence of studies from African settings |
| Conclusions | 21 | Provide a general interpretation of the results with respect to the review questions and objectives, as well as potential implications and/or next steps. | **Reported on page 33**  PBL appears beneficial, but more research is needed to explore long-term effects and adaptations among first-year undergraduate students in low-resource settings |
| **FUNDING** | | | |
| Funding | 22 | Describe sources of funding for the included sources of evidence, as well as sources of funding for the scoping review. Describe the role of the funders of the scoping review. | **Reported on page 34**  No funding was received for this study |

JBI = Joanna Briggs Institute; PRISMA-ScR = Preferred Reporting Items for Systematic reviews and Meta-Analyses extension for Scoping Reviews.

* Where *sources of evidence* (see second footnote) are compiled from, such as bibliographic databases, social media platforms, and Web sites.

† A more inclusive/heterogeneous term used to account for the different types of evidence or data sources (e.g., quantitative and/or qualitative research, expert opinion, and policy documents) that may be eligible in a scoping review as opposed to only studies. This is not to be confused with *information sources* (see first footnote).

‡ The frameworks by Arksey and O’Malley (6) and Levac and colleagues (7) and the JBI guidance (4, 5) refer to the process of data extraction in a scoping review as data charting*.*

§ The process of systematically examining research evidence to assess its validity, results, and relevance before using it to inform a decision. This term is used for items 12 and 19 instead of "risk of bias" (which is more applicable to systematic reviews of interventions) to include and acknowledge the various sources of evidence that may be used in a scoping review (e.g., quantitative and/or qualitative research, expert opinion, and policy document).

*From:* Tricco AC, Lillie E, Zarin W, O'Brien KK, Colquhoun H, Levac D, et al. PRISMA Extension for Scoping Reviews (PRISMAScR): Checklist and Explanation. Ann Intern Med. 2018;169:467–473. [doi: 10.7326/M18-0850](http://annals.org/aim/fullarticle/2700389/prisma-extension-scoping-reviews-prisma-scr-checklist-explanation).
