# Supplementary material for: The effects of problem-based learning (PBL) on undergraduate medical students’ critical thinking and communication skills development: A scoping review across resource-rich and resource-limited settings (2015–2024)
Source: PLoS One. 2026 Feb 13;21(2):e0342599. doi: 10.1371/journal.pone.0342599 (PMC12904385; doi:10.1371/journal.pone.0342599)
Supplement: Attachment — Submitted filename: Response to Reviewers.docx [file pone.0342599.s003.docx]

Rebuttal Letter

Response to Reviewers on the Manuscript ID: PONE-D-25-14527

Introduction

We appreciate the Academic Editor and reviewers for the time taken to review our manuscript. We have revised the manuscript as needed and respond to each comment below. Reviewer #1's comments were excluded as editorially instructed.

It is our believe that this will go a long way to improve the readability and quality of the paper.

We now proceed to respond to the reviewers’ comments

Reviewer #2 Comments

Major Comment 1: PRISMA flow chart is not clear. The reason for the second exclusion is not provided.

Response**:** We have revised the PRISMA flow diagram to detail reasons for exclusions at each stage of the selection process.

Major Comment 2: References need to be reformatted according to journal style.

Response: All references have been reformatted according to PLOS ONE style guide, and typographical errors have been corrected.

Minor Comment 1: Define TBL.

Response**:** We have now defined TBL (Team-Based Learning) at first mention in the manuscript. “Is a version of PBL, where students work in teams to apply their knowledge through activities to solve problems and improve their understanding”

Minor Comment 2: Table 1 formatting issue.

Response**:** The format of Table 1 has been adjusted for improved readability in both Word and PDF.

Minor Comment 3: Language issues (e.g., "cannot established").

Response: The grammatical issues have been reviewed and edited. Now replaced with “cannot establish”

Reviewer #3 Comments

Comment 1**:** Why have so few papers been included? Some of the papers meeting the criteria can be easily obtained by Google Scholar.

Response**:**

We appreciate the reviewer’s observation. “As outlined in the Methods section, we applied clearly defined inclusion and exclusion criteria to guide our screening process. While the initial search yielded a substantial number of articles, the majority were excluded for reasons such as focus on postgraduate populations, non-English language, non-medical disciplines, lack of direct measurement of critical thinking or communication, or absence of empirical data. Only studies involving undergraduate medical students, published in English, directly addressing critical thinking or communication, and employing empirical methods were retained. We acknowledge that the resulting number of included studies is relatively small. However, the application of these stringent criteria was necessary to ensure methodological rigor and thematic relevance and the objective of the scoping review was to map existing evidence to inform a full-blown systematic review. However, we have acknowledged as a limitation and this has been duly addressed as a newly added subsection within the Discussion”.

Comment 2: The small sample does not justify the strength of conclusions.

Response: This has been noted as a limitation and the conclusion has been revised accordingly to meet the limits of the data available.

Comment 3: Language: "trials," "causation," "guaranteed," etc. are not suitable.

Response: These words have been changed throughout the manuscript to better reflect the descriptive, non-experimental nature of a scoping review. Now changed to, “studies, may support, however causation has been removed”.

Line-by-line language issues ("monumental proportions," "time immemorial," "showcases," etc.)

Response: These have been revised or removed to ensure precision and academic tone throughout the manuscript. Monumental proportions removed, time immemorial is now replaced with “in time past” and showcase now replaced with “demonstrates”

Line-by-Line Comments and Responses

Lines 30 and 33:

The reviewer expressed concern that major conclusions were being drawn from a single study. In response, we have revised the Abstract and Results sections to clarify that findings from that study are preliminary and indicative. Now revised to “As borne out by analyses of the other included studies, PBL **may** enhance critical thinking and communication across all levels and in both resource-limited and resource-rich educational environments**. Evidence suggests** that PBL may supports the development of critical thinking and communication skills in first-year medical students. Further research is required to analyze long-term effects and adaptation challenges in low-resource settings”.

Line 46:

The phrase “lots of” was considered vague. We have addressed this by replacing it with more specific language: “Many pedagogical strategies such as competency-based education, problem-based learning, and simulation-based training…”, which improves clarity and precision.

Line 47:

The phrase “time immemorial” was noted to be hyperbolic and historically inaccurate. This expression has been removed from the revised manuscript. The sentence now reads ‘’ In time past“

Line 54:

The reviewer noted that the citation did not fit the assertion. Upon review, we discovered that the citation was mistakenly listed as Cooke et al., 2010 when the correct reference is “Cooke et al., 2006”. This has now been corrected in both the in-text citation and the reference list, ensuring alignment with the claim about the Flexner Report and its impact.

Line 61:

The reviewer highlighted that Barrows described PBL as a pedagogical philosophy rather than a method. We have revised this section to reflect Barrows’ original stance accurately, emphasizing the flexible and varied nature of PBL implementation. Now revised to “Barrows (1986) emphasized that PBL is not a fixed method but rather a pedagogical philosophy, noting the wide variety of objectives and approaches encompassed under the PBL umbrella”.

Line 68:

The word “augusted,” which is not standard English, has been removed from the manuscript.

Line 72:

The phrase “monumental historical proportions” was considered vague. This language has been removed or rephrased to maintain academic tone. Now reads “Over time, PBL reached widespread adoption within the medical education from teacher-centered approach to student-centered self-directed instruction,”

Line 81:

The reviewer noted an organizational issue. We have restructured this paragraph so that it flows more logically from the previous section, improving cohesion.

Lines 83–85:

The reviewer asked whether the statement referred to curricula in Ghana or generally. We have clarified that this refers specifically to Ghanaian medical curricula and have updated the text accordingly. Now rephrased to “For this reason, medical curricula in Ghana, including those at the University for Development Studies and the University of Cape Coast, have adopted PBL”

Line 90:

The reviewer suggested adding references to Garrison (1991) and Newman et al. (1995). Both citations have now been included alongside Facione (2011) to strengthen the theoretical foundation of the section on critical thinking assessment. Now revised to “

Lines 101–102:

The reviewer pointed out the need to distinguish between self-reported data and assessed outcomes. This distinction has been made throughout the manuscript, particularly in the Limitations and Results sections.

Line 105:

An organizational concern was raised about the introduction of a new topic. We have revised the paragraph structure to improve clarity and flow between topics.

Line 115:

The use of Team-Based Learning (TBL) was questioned in this context. We have clarified that TBL was discussed as a variant of PBL and included appropriate justification and references to support its relevance to communication skills development.

Lines 130–131:

The reviewer asked whether a specific point was addressed later in the manuscript. This has been clearly elaborated in the Results section, making the connection explicit.

Lines 163–164:

The reviewer asked where the study selection process was presented. We have revised the text to state: “as shown in figure 1”, which presents the identification, screening, and selection of studies.”

Lines 224–225:

The reviewer questioned whether rich and poor settings were the only thematic trends? We have clarified in both the Results and Discussion sections that while thematic differences were observed, contextual factors such as infrastructure, faculty training, and digital tools were also considered.

Lines 235–236:

The term “rigorous process” was not clearly defined. We have expanded the description of our selection process, provided justification for excluded studies, and referenced the PRISMA flowchart to enhance transparency.

Line 242:

The term “showcases” was considered too informal. We have replaced this term with more academic alternatives such as “demonstrates” throughout the manuscript.

Lines 310–313:

The reviewer noted that heterogeneity in data limits generalizability. This limitation has been explicitly acknowledged in both the Discussion and Limitations sections.

Lines 329–333:

The reviewer noted that the small sample size does not support broad claims. We have revised our claims to reflect the preliminary nature of the findings and clarified that the study suggests trends rather than drawing definitive conclusions.

Lines 365–366:

Assertions based on limited data were noted as overstatements. These have been revised to a more tentative tone throughout the manuscript.

Lines 409–410:

The reviewer questioned the clarity of statement “Nonetheless, it is clear that the application of nearly all structured PBL activities, such as role-409 playing, debates, and expert-facilitated discussions, take place in well-resourced environments.”. We have now supported it with additional explanation and contextualization to clarify the point. Now revised to “While PBL has been applied in all sorts of educational contexts, structured activities such as role-plays, debates, and expert discussions are most often conducted in environments with access to resources. This has been positively evidenced by four of the studies reviewed of each which recorded positive gains in the communication and reasoning skills of students”

Line 424:

The reviewer asked where guidance for students might come from. We have addressed this by revising it to “curriculum committees, national accreditation bodies, and faculty development initiatives as potential sources of structured guidance”.

Line 432:

The reviewer noted that causation cannot be inferred. We have revised relevant sections to clarify that the included studies are descriptive or cross-sectional and do not establish causality.

Line 437:

The use of the term “trials” was flagged as inappropriate in this context. All such references have been replaced with “studies” .

Line 445:

The phrase “research gaps are filled up” was deemed incorrect. We have rephrased this to indicate that the study helps to “address” existing research gaps.

Line 447:

The claim of “guaranteeing” an outcome was seen as too strong. We have reworded this section to reflect the inherent uncertainties and limitations in the data. Now revised to “could also improve as well”

We have also amended the Data Availability Statement to**:**

"All data are in the manuscript and/or supporting information files."

We hope the revised manuscript now fulfills the requirements of the reviewers and the journal. We appreciate the constructive feedback, which has improved the paper.

Sincerely,

Bruce Ayabilla Abugri

On behalf of all authors.

Senior Research Assistant,

University for Development Studies,

School of Medicine.
