# Supplementary material for: The effects of problem-based learning (PBL) on undergraduate medical students’ critical thinking and communication skills development: A scoping review across resource-rich and resource-limited settings (2015–2024)
Source: PLoS One. 2026 Feb 13;21(2):e0342599. doi: 10.1371/journal.pone.0342599 (PMC12904385; doi:10.1371/journal.pone.0342599)
Supplement: Attachment — Submitted filename: Rebuttal Letter.docx [file pone.0342599.s004.docx]

**Subject: Response to Reviewers' Comments on Manuscript ID**: **PONE-D-25-14527R2**

Title: The effects of problem-based learning (PBL) on undergraduate medical students' critical thinking and communication skills development. A scoping review across resource-rich and resource-limited settings (2015-2024).

Dear Editor,

Thank you for the opportunity to revise and resubmit our manuscript. We are grateful to you and the reviewers for the constructive and insightful feedback provided. We have carefully considered all comments and have made substantial revisions to address them. We believe the revised manuscript is now significantly improved in terms of clarity, methodological rigor, and practical relevance.

Below, we provide a point-by-point response to each of the comments raised. All changes in the manuscript have been highlighted using the "Track Changes" feature for your convenience.

Response to Editor's Comments

**Comment 1**: "Please explain in more depth how and why the initial 20,789 papers were excluded"

**Response:** Thank you for this important request for methodological transparency. We have now added a detailed paragraph in the Results section that explicitly outlines the reasons for exclusion at both the title/abstract screening stage and the full-text review stage. We acknowledge that this value 20,789 was mistakenly recorded instead of 212 and we have now corrected it. This provides a clear, step-by-step account of the screening process, including the primary reasons for exclusion (e.g., wrong population, intervention, outcome, publication type). This addition can be found on page **[10-11]**, and **lines [211-232]**.

**Comment 2**: "If there is no ability to synthesize or analyze, then what can we learn from this effort? I would suggest... find a way to analyze the papers you did include qualitatively or thematically... Braun, V., & Clarke, V. (2006)."

**Response**

We appreciate this valuable suggestion. Our original wording may have unintentionally implied that we did not conduct any synthesis. We have now explicitly described the descriptive thematic synthesis of the included studies, drawing on the framework proposed by Braun and Clarke (2006) and Nowell et al. (2017). This is detailed in the Methods section under subheading Synthesis of results on page (**10)** and lines **(200-207)** with a new section titled thematic synthesis of result added. To clarify, while heterogeneity in study design, sample size, and assessment tools precluded quantitative or inferential statistical synthesis, this is consistent with the aims of a scoping review (Arksey & O’Malley, 2005; Tricco et al., 2018). We have revised the manuscript to reflect this.

**Response to Reviewer #4's Comments**

We thank Reviewer #4 for their thoughtful engagement with our manuscript.

**Comment 1:** "The specific reasons for exclusion at each stage remain unclear, making it difficult to assess the comprehensiveness of the review."

**Response:** We agree that this was a critical point to clarify. We have now added a detailed description in the results section that explicitly outlines the reasons for exclusion at both the title/abstract screening stage and the full-text review stage. Also, we have revised the PRISMA diagram appropriately to reflect this new addition. This provides a clear, step-by-step account of the screening process, including the primary reasons for exclusion. This addition can be found on pages **[11-12], lines [211-232].**

**Comment 2:** "These methodological variations... make it challenging to synthesize findings meaningfully."

**Response:** We acknowledge the inherent methodological limitations of the primary studies. Our adoption of a thematic analysis was specifically chosen to address this challenge. By synthesizing the data thematically rather than statistically, we were able to identify meaningful patterns and insights across heterogeneous studies. We have also been more explicit in acknowledging these limitations in the Discussion section, framing our findings as tentative but valuable pointers for future research.

**Comment 3**: "The abstract and conclusion make claims about PBL 'may support the development...' and suggest 'effectiveness,' which goes beyond the stated objective..."

**Response:** Thank you for this important correction. We have carefully revised the language throughout the manuscript, particularly in the Abstract and Conclusion, to ensure our claims are appropriately cautious and consistent with the exploratory nature of a scoping review. **We now use phrases such as "****may be associated with perceived improvements" and "****findings remain tentative," which more accurately reflect our findings.**

**Comment 4:** "...limited in-depth thematic analysis... influence of 'cultural backgrounds'... is not systematically examined... unclear how medical educators can practically apply these findings."

**Response**: This was an excellent point, and we have made significant additions to address it. First, we have added a new subsection in the Discussion titled "Influence of cultural and diverse educational backgrounds” to systematically examine this crucial factor **see pages (23-24) and lines (455-475**). Second, and most importantly, we have added a dedicated major section titled "Practical implications for educators implementing PBL in different resource settings **see pages (26-27) and lines (517-540)**." This new section translates our findings into actionable advice for educators, providing specific guidance for resource-rich, resource-limited, and culturally diverse contexts.

**Response to Reviewer #5's Comments**

We thank Reviewer #5 for their valuable suggestions, which have helped us refine the manuscript.

**Comment 1:** "Suggest to revise the keyword first year medical student..."

**Response:** We have revised the keyword list as suggested to include "first year medical student" for better indexing.

**Comment 2**: "The introduction... is long-winded... a disconnect between the history of PBL and twenty-first-century education."

**Response:** We have streamlined the Introduction to create a more direct narrative link between the historical context of medical education and the contemporary challenges faced by first-year students in different resource settings, thereby better framing the rationale for our study.

**Comment 3:** "The definition for critical thinking needs and communications skills need to be clarified..."

**Response:** We have clarified the operational definitions of both critical thinking and communication skills in the Introduction, explicitly linking them to their measurable indicators (e.g., analytical reasoning, problem-solving and collaborative skills) to ensure conceptual precision at Pages **(4-5)** and lines (**87-95).**

**Comment 5**: "Table 1: Data extraction text is unclear."

**Response:** We have reformatted Table 1 to improve its clarity and readability, ensuring the data is presented in a professional and unambiguous manner.

**Comment 6**: "For the results section, there happens to be a conflict on generic skills..."

**Response:** We have standardized the terminology for "generic skills" throughout the manuscript (inclusion criteria, Table 1, and main text) to resolve the noted inconsistency. The definition is now consistently presented as **“(analytical reasoning, problem-solving and collaborative skills)."**

**Comment 7**: "The discussion was rather limited to outdated citations..."

**Response:** We have updated the Discussion, particularly in the newly added sections, with more current literature (including citations from 2022-2024) to ensure our arguments are situated in the most recent scholarly context. Also, the scoping review was conducted in 2024, and inclusion criteria included studies from 2015 to 2024, hence the discussion featured studies from 2015 upwards.

Once again, we thank you and the reviewers for your time and expertise. We believe the revised manuscript is now a much stronger contribution to the literature and hope that it is now suitable for publication in PLOS ONE.

Sincerely,

Victor Mogre

Corresponding Author

Department of Health Professions Education and Innovative Learning, School of Medicine, University for Development Studies, Tamale, Ghana.

Email: vmogre@uds.edu.gh
