# Supplementary material for: The effects of problem-based learning (PBL) on undergraduate medical students’ critical thinking and communication skills development: A scoping review across resource-rich and resource-limited settings (2015–2024)
Source: PLoS One. 2026 Feb 13;21(2):e0342599. doi: 10.1371/journal.pone.0342599 (PMC12904385; doi:10.1371/journal.pone.0342599)
Supplement: Attachment — Submitted filename: Response_to_Reviewers_auresp_3.docx [file pone.0342599.s005.docx]

Dear Editor,

Thank you for your careful review of our scoping review manuscript and for the clear, actionable comments. I have revised the manuscript accordingly and outline below how each point has been addressed.

(a) Arrange your manuscript into numbered sections and sub-sections. Ensure consistency across font styles etc.

**Response**: We have reorganised the manuscript into clearly numbered sections and sub-sections using a consistent heading hierarchy. I also standardised formatting across the document, including font type, font size, heading styles, spacing, and paragraph formatting, to ensure uniform presentation throughout.

(b) Make sure all the tables and figures are titled and start at the top of the page, where possible.

**Response:** We ensured that all tables and figures are sequentially numbered and provided with clear, descriptive titles/captions. I also adjusted placement so that each table and figure begins at the top of a page.

(c) Add a subsection under “Introduction” that provides a working definition of PBL for your study. Describe what PBL is and what sets it apart from other forms of active learning such as case-based learning, inquiry learning, etc.

**Response**: we have added a dedicated subsection under the Introduction presenting a working definition of PBL used in this review. In that subsection, we describe the essential features of PBL and explicitly differentiate it from case-based learning and inquiry learning, with a supporting citation.

(d) Under the “Limitations” sub-section, address the absence of the ERIC database in your scoping review.

**Response**: ERIC (Education Resources Information Center) was searched. The search retrieved records; however, none was related to the study objectives and inclusion and exclusion criteria after titles screening. We have updated the manuscript to (i) list ERIC under the information sources, search strategy and the PRISMA-ScR flow diagram, rather than presenting ERIC as an omitted database.

**Response to reviewers**

Thank you for the opportunity to revise the manuscript further. We have carefully addressed the additional concerns raised by Reviewer #6 and Reviewer #7. Below, we respond point-by-point and indicate where the manuscript has been strengthened.

**Response to Reviewer #6**

Comment: The search strategy was not comprehensive. The authors used only “problem-based learning,” excluding two important synonyms (case-based learning and inquiry-based learning). ERIC was not included.

**Response**: We agree that the search strategy must be transparent and defensible. However, we do not treat case-based learning and inquiry-based learning as direct synonyms of PBL because they represent distinct instructional approaches. This is now explicitly reflected in the manuscript’s working definition of PBL, where PBL is defined by its core features and distinguished from related active learning approaches.

**Regarding ERIC**: ERIC was included as an information source and is now consistently reported in the Methods section under “Information sources”,” search strategy” I also updated the study selection narrative and PRISMA-ScR flow reporting to reflect the ERIC search yield and its screening outcome. This corrects the earlier presentation of ERIC in the flow diagram text and ensures alignment between Methods and Results.

**Response to Reviewer #7**

Comment 1: Fix the in-text citation style. Remove the comma before numbering at the end of each sentence.

**Response:** We revised the in-text citation punctuation throughout the manuscript to match the journal’s numeric citation conventions. Specifically, I removed punctuation patterns such as “..., (33, 34)” and applied consistent formatting across sentences. This issue was present in the Results/Discussion narrative and has now been corrected.

Comment 2: Several citations are too old (e.g., 1986). Please provide updated citations.

**Response:** We understand the concern. Where older citations were used to acknowledge seminal origins (e.g., foundational PBL descriptions), We retained them only when they served a clear historical or definitional purpose and paired them with contemporary sources that reflect current evidence and implementation realities. For example, the manuscript now anchors PBL discussion with more recent syntheses and empirical summaries already included in the reference list (e.g., more recent reviews and meta-analytic work) while keeping seminal citations sparingly for conceptual lineage.

**Comment 3**: Elaborate on resource-rich and resource-limited settings. What resources are needed for PBL? Give examples of PBL conducted in resource-rich and resource-limited settings.

**Response**: We expanded the “resource-rich vs resource-limited settings” sections to be more explicit about what “resources” mean in PBL implementation and how these resources plausibly shape delivery. We clarified that “resources” include, at minimum: trained facilitators, protected small-group time, appropriate tutorial spaces, access to learning materials (library and internet access where relevant), and support for case design and formative feedback. We also provided concrete examples drawn from the included studies, contrasting settings described as better resourced (e.g., Saudi Arabia and China) with settings described as more resource constrained (e.g., Pakistan, India, and Nepal), while keeping the interpretation tied to the evidence base mapped in this review.

In addition, I strengthened the practical implications section to make the resource distinctions actionable for educators, including examples of how PBL can be supported in each context (technology-supported implementation in better resourced settings versus pedagogy-led implementation and peer support structures in constrained settings).

Thank you again for these specific comments. I believe the revisions improve methodological clarity, citation accuracy, and interpretive precision, while keeping the manuscript aligned with its stated scope and eligibility criteria.

Sincerely,

Bruce Ayabilla Abugri
